# Unveiling the Enigma of Post-implantation Syndrome Following Aortic Dissection Repair: A Case Report

**DOI:** 10.7759/cureus.38928

**Published:** 2023-05-12

**Authors:** Prachi Yadav, Aman Agrawal, Sanket S Bakshi, Richa Chaudhary

**Affiliations:** 1 Department of Medicine, Jawaharlal Nehru Medical College, Datta Meghe Institute of Higher Education and Research, Wardha, IND; 2 Department of Pediatrics, Jawaharlal Nehru Medical College, Datta Meghe Institute of Higher Education and Research, Wardha, IND

**Keywords:** inflammatory marker, inflammation, fever, aortic dissection, post-implantation syndrome

## Abstract

Post-implantation syndrome (PIS) is a frequent complication after aortic dissection repair surgery, posing significant risks to patient recovery and survival. We present a case report of a 62-year-old male who underwent aortic dissection repair surgery and developed PIS. The patient exhibited symptoms of fever, pain, and inflammation at the surgery site, along with increased levels of inflammatory markers. He was managed with a combination of anti-inflammatory medications, pain management, and antibiotics, which gradually improved symptoms over weeks. Our case highlights the importance of recognizing the potential for PIS in patients undergoing aortic dissection repair surgery and employing timely interventions to manage this condition.

## Introduction

As per the European Society of Cardiology Task Force and European cardiovascular mortality and morbidity data, cardiovascular illnesses constitute a significant cause of mortality in the majority of industrialized and developing nations. The significantly high cardiovascular mortality rate is due to aortic diseases. Acute aortic dissection is a potentially fatal condition and very rare, with a one percent death rate every hour if left untreated. According to the location and extent of aortic involvement, aortic dissection has been classified as A type or B type by the Stanford classification [1]. The prognosis of acute aortic dissection has greatly enhanced with advancements in medical and surgical interventions [2]. Chest discomfort, emesis, diaphoresis, syncope, stroke, hypertension, cardiac failure, shock, and sudden cardiac arrest are all symptoms and signs of aortic dissection [3].

Furthermore, fever may be prolonged in some acute cases, making it difficult to evaluate and confirm the relation to acute aortic dissection. Similar issues were reported, correlating the unexplained febrile episodes with acute aortic dissection. Post-implantation syndrome (PIS) was initially registered as a fever and leucocytosis syndrome following the implantation of a stent graft in the aorta. The etiology of PIS is due to the attribution of endovascular reconstruction to the systemic inflammatory reaction. There is a release of various inflammatory markers like tumor necrosis factor alpha (TNF-α) and interleukin-6 (IL-6). This inflammatory response could be triggered by endothelial dysfunction, indicating a synergistic relationship between graft material and endovascular surgical approach [4]. The outcome of PIS in high-risk patients (elderly with co-morbidities) is unknown despite the patients' good tolerance. Fatigue and fever are symptoms of PIS, which are correlated to an upsurge in inflammatory biomarkers [5]. The type of inflammatory markers and their cut-off values are still up for contention. In the literature, the triad consisting of fever, leucocytosis, and increased C reactive protein (CRP) is usually used to define PIS. Researchers used criteria of systemic inflammatory response syndrome, fever, and leucocytosis, which were considered crucial indicators [6].

## Case presentation

**Figure 1:**
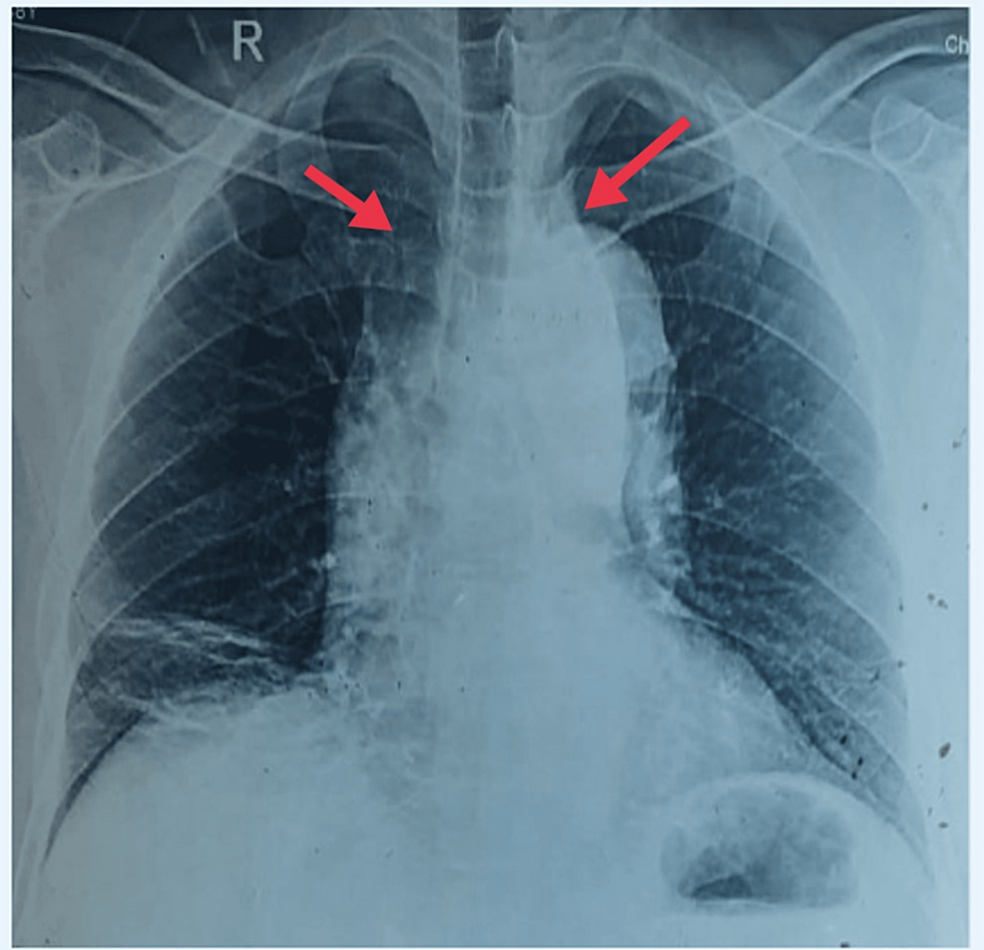
Chest X-ray showing superior widened mediastinum

A 62-year-old male patient with a history of hypertension presented to the emergency department with complaints of chest pain and shortness of breath which is of sudden onset and aggravated by exertion associated with sweating and palpitations in the past two hours. A general examination of the patient suggested that he was afebrile on touch, pulse rate was 98/minute, blood pressure in the right-sided arm in the supine position was 190/130mm Hg, the left arm was 180/120mm Hg, respiratory rate was 34/minute abdomino-thoracic type, and pallor was present on examination. Cardiovascular, respiratory, and central nervous system examinations revealed normal findings. Chest X-ray revealed a superior widened mediastinum (Figure 1).

**Figure 2:**
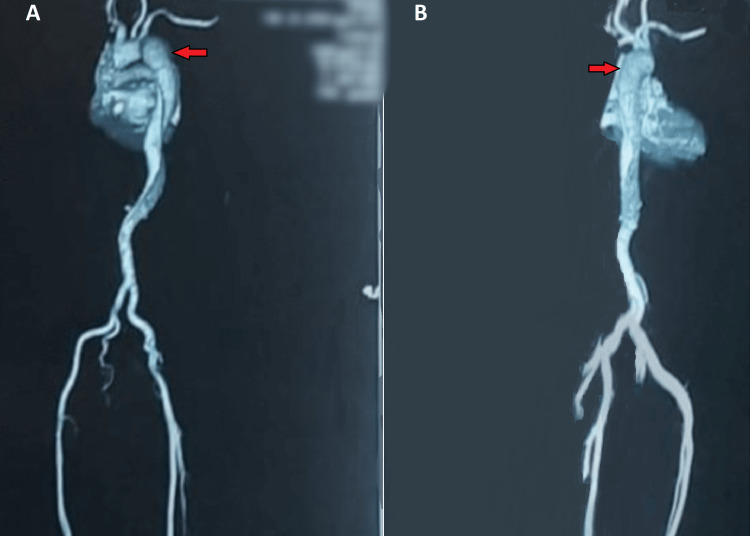
(A) CT abdominal angiography anteroposterior view showing aortic dissection distal to the left subclavian artery; (B) CT abdominal angiography lateral view showing aortic dissection

ECG revealed normal sinus rhythm with no evidence of myocardial ischemia and hematological investigation was normal. Considering the possibility of aortic dissection, an emergency CT aortogram was done which revealed mild dilatation of the proximal descending aorta with an internal intimal flap which involves descending aorta just to the origin of the left subclavian artery favoring type B Stanford aortic dissection and extending to involve thoracic aorta to abdominal descending aorta just close to the diaphragm (Figure 2).

In view of the type B aortic dissection, he was started on beta-blocker (intravenous labetalol followed by oral long-acting metoprolol). With this, his BP decreased to 130/90mmHg and his pain also started to decrease. A detailed history was taken at this point, revealing a similar disease in his father, who died of the disease. Hence, the possibility of a genetic cause of aortopathy was kept as a possibility and was planned to be evaluated. But the next morning, he developed a sudden severe tearing type of chest and back pain and developed hypotension and tachypnea, tachycardia with bilateral crepts, and hypoxia. There was an early diastolic murmur at the base of the heart suggestive of aortic regurgitation. A bedside echo was done, which revealed aortic dissection from the aortic root extending across the arch into the descending aorta. There was also severe aortic regurgitation. Considering this patient was immediately taken for surgical repair, an intraoperative dissection flap was noted extending from the aortic valve to the descending aorta, close to the diaphragm. Using the frozen elephant trunk graft technique, aortic reconstruction was done, which involved the reimplantation of neck vessels into the arch and aortic bioprosthetic valve replacement as well. Postoperatively, on day three, the patient developed febrile episodes along with pain close to the surgical site. A possibility of surgical site infection was considered, and after sending labs like complete blood counts, procalcitonin, CRP, blood culture, and wound swab culture, antibiotics were escalated to meropenem, amikacin, and vancomycin to cover hospital-acquired infections. Laboratory investigations showed an elevated white blood cell count and C-reactive protein levels. There was leukocytosis (total leukocytes count (TLC) 15000/cumm) and CRP was elevated (25 mg/dl) but repeated blood cultures, urine cultures, and wound swabs were negative. Despite antibiotics, the fever persisted. D-dimer was elevated. Considering this, PIS was diagnosed based on the patient's clinical presentation and laboratory findings. Antibiotics were decreased, and the patient was started on anti-inflammatory medications. The anti-inflammatory regimen consisted of high-dose steroids and nonsteroidal anti-inflammatory drugs (NSAIDs).

The patient's symptoms gradually improved over the course of the next two weeks. Followup laboratory investigations showed decreased inflammatory markers (TLC 8000/cumm and CRP 1.5mg/dl), and the patient's fever subsided. He was eventually discharged from the hospital and advised to continue with outpatient follow-up. Hence, the clinical picture and lab reports were against the infectious etiology, and the diagnosis of PIS was proven as not only the clinical status but also the inflammatory markers improved with anti-inflammatory treatment despite discontinuation of antibiotics.

## Discussion

PIS is a notorious complication following aortic dissection repair surgery that can lead to fatality. PIS can have varied presentations, including fever, inflammation, and leukocytosis, which can significantly impact the patient's recovery. The exact cause of PIS remains unclear, but it is assumed to be associated with the inflammatory response after surgery.

Several studies have investigated the incidence of PIS after aortic dissection repair surgery. In a retrospective study of 38 patients who underwent a frozen elephant trunk procedure, the incidence of PIS was 44.7% [7]. Another study of 74 patients who underwent thoracic endovascular aortic repair (TEVAR) reported an incidence of PIS of 22.97% [8]. A more recent study of 149 patients found an incidence of PIS of 39% [9]. The wide range of reported incidence rates can be attributed to differences in patient populations and surgical techniques used in these studies. Ibrahim et al. mentioned in their study that PIS occurred in patients who underwent open frozen trunk procedures for type A dissection and ascending aortic aneurysms [7]. Volevski et al. [8] have reported in their study that PIS occurred following TEVAR. The aortic pathologies treated in their studies were aortic dissection type B, abdominal aortic aneurysm, and perforated aortic ulcers. The above studies suggest that PIS is a common occurrence after aortic therapies. The type of aortopathy (dissection vis e vis aneurysm) and the type of management (endovascular aneurysm repair (EVAR) vis e vis TEVAR) have an impact on the occurrence of PIS, but it can occur in all the subtypes. As the initial suspicion is of post-surgical infection, the patient is wrongly treated for many days without improvement. Hence, it is imperative to keep the possibility of PIS in mind in these cases to avoid unnecessary treatment and bring about recovery with proper treatment.

Various treatment strategies have been proposed to manage PIS, including anti-inflammatory drugs, antibiotics, and pain management. The combination of high-dose steroids and NSAIDs has been shown to be effective in reducing inflammation and pain associated with PIS [10]. The use of antibiotics is recommended in cases of suspected infection, which can exacerbate the inflammatory response. In severe cases, surgical intervention may be required to drain abscesses and remove infected tissue.

Several preventive measures have been suggested to reduce the risk of developing PIS. Minimally invasive surgical techniques have been shown to reduce the incidence of PIS. Optimizing post-operative care, such as early mobilization and respiratory physiotherapy, can also help prevent PIS [11].

## Conclusions

PIS is a common complication after aortic dissection repair surgery and should be considered in patients who develop fever and evidence of inflammation, especially when infection after the surgery has been ruled out. Early recognition and management of PIS can significantly improve clinical outcomes and reduce morbidity and mortality. Further research is necessary to develop more effective strategies for preventing and managing PIS.

## References

[1] Arnaoutoglou E, Papas N, Milionis H, Kouvelos G, Koulouras V, Matsagkas MI (2010). Post-implantation syndrome after endovascular repair of aortic aneurysms: need for postdischarge surveillance. Interact Cardiovasc Thorac Surg.

[2] Velazquez OC, Carpenter JP, Baum RA, Barker CF, Golden M, Criado F (1999). Perigraft air, fever, and leukocytosis after endovascular repair of abdominal aortic aneurysms. Am J Surg.

[3] Arnaoutoglou E, Kouvelos G, Milionis H (2011). Post-implantation syndrome following endovascular abdominal aortic aneurysm repair: preliminary data. Interact Cardiovasc Thorac Surg.

[4] Voûte MT, Bastos Gonçalves FM, van de Luijtgaarden KM, Klein Nulent CG, Hoeks SE, Stolker RJ, Verhagen HJ (2012). Stent graft composition plays a material role in the postimplantation syndrome. J Vasc Surg.

[5] Dosluoglu HH, Lall P, Blochle R, Harris LM, Dryjski ML (2014). Ambulatory percutaneous endovascular abdominal aortic aneurysm repair. J Vasc Surg.

[6] Gabriel EA, Locali RF, Romano CC, Duarte AJ, Palma JH, Buffolo E (2007). Analysis of the inflammatory response in endovascular treatment of aortic aneurysms. Eur J Cardiothorac Surg.

[7] Ibrahim A, Marchiori E, Eierhoff T (2021). Post-implantation syndrome after frozen elephant trunk is associated with the volume of new-onset aortic thrombus. J Thorac Dis.

[8] Volevski LA, Vasiloi I, Abudureheman N (2023). Impact of the underlying aortic pathology on postimplantation syndrome after endovascular thoracic aortic repair. J Cardiovasc Surg (Torino).

[9] Soares Ferreira R, Oliveira-Pinto J, Ultee K (2021). Long term outcomes of post-implantation syndrome after endovascular aneurysm repair. Eur J Vasc Endovasc Surg.

[10] Chamkouri N, Absalan F, Koolivand Z, Yousefi M (2023). Nonsteroidal anti-inflammatory drugs in viral infections disease, specially COVID-19. Adv Biomed Res.

[11] Jacob P, Gupta P, Shiju S (2021). Multidisciplinary, early mobility approach to enhance functional independence in patients admitted to a cardiothoracic intensive care unit: a quality improvement programme. BMJ Open Qual.

